# Differentiation defects reposition sebaceous glands as inflammatory instigators in the early pathogenesis of hidradenitis suppurativa

**DOI:** 10.3389/fimmu.2026.1785747

**Published:** 2026-06-08

**Authors:** Xiang Chen, Jiaqi Li, Yibo Feng, Zhanyan Pan, Qiong Wu, Xinyi Xu, Qian Zhao, Tingting Hu, Guangjie Chen, Christos C. Zouboulis, Xiaohui Mo, Qiang Ju

**Affiliations:** 1 Department of Dermatology, Renji Hospital, Shanghai Jiaotong University School of Medicine, Shanghai, China; 2 Department of Immunology and Microbiology, School of Medicine, Shanghai Jiaotong University, Shanghai, China; 3 Department of Dermatology, Venereology and Immunology, Universitaetsklinikum Ruppin-Brandenburg, Brandenburg Medical School Theodor Fontane and Faculty of Health Sciences Brandenburg, Neuruppin, Germany

**Keywords:** Hidradenitis suppurativa, lysophosphatidylcholine, sebaceous gland, sebum, tight junction

## Abstract

**Background:**

The role of sebaceous glands (SGs) in the early pathogenesis of hidradenitis suppurativa (HS) remains undefined, with an unclear causal relationship to inflammatory sequelae. The aim of this study is to determine whether SG aberrations constitute a primary pathogenic driver in early HS and elucidate the underlying mechanisms.

**Methods:**

We employed histology, single-cell RNA sequencing, and *in-vitro* functional assays. Clinical specimens included non-lesional skin (NLS, *n* = 6), early lesional skin (LS, Hurley I, *n* = 12) from HS patients, and healthy controls (HC, *n* = 8). Human SZ95 sebocytes were used for mechanistic studies, including gene knockdown, bulk RNA sequencing, and lipidomic analysis.

**Results:**

SG size was significantly reduced in both NLS (47.26% of HC area, *p* = 0.04) and LS (30.74%, *p* = 0.006). Single-cell RNA sequencing analysis revealed aberrant stem cell commitment in the hair follicle junctional zone and a significant downregulation of tight junction signaling (e.g., CLDN1, TJP1, OCLN) in HS SGs, associated with compromised barrier integrity and early immune cell (CD45^+^) infiltration. CLDN1 knockdown in SZ95 sebocytes recapitulated these findings, inducing a robust pro-inflammatory response (upregulation of IL-1β, TNF-α, IL-6, S100A7/A8, and CXCL8), suppression of the sebocyte lineage regulator c-Myc, and a shift toward keratinocyte-like differentiation. This was accompanied by metabolic reprogramming, specifically overproduction of lysophosphatidylcholine (LPC). Exogenous LPC directly promoted proliferation and inflammatory cytokine secretion in HaCaT keratinocytes.

**Conclusions:**

SG dysfunction, initiated by tight junction disruption and CLDN1 deficiency, is a primary event in early HS. This leads to aberrant sebocyte differentiation, inflammatory amplification, and sebum metabolite LPC-mediated crosstalk with keratinocytes. Our findings position SG-derived LPC as a potential novel biomarker and therapeutic target in HS pathogenesis.

## 1 Introduction

Hidradenitis suppurativa (HS) is a chronic inflammatory dermatosis characterized by deep-seated nodules, abscesses, sinus tracts, and fibrotic scarring predominantly affecting intertriginous regions, including axillary, inguinal, and perianal areas (1). Current evidence supports a multifactorial pathogenesis that involves the interplay between genetic susceptibility (notably γ-secretase component gene variation) and environmental risk factors such as smoking and obesity. Although the precise mechanism of HS partially remains to be elucidated, follicular hyperkeratosis, occlusion of the pilosebaceous-apocrine unit, and perifollicular inflammation are considered to be primary events during the initiation and aggravation of the disease (2).

In recent years, a significant association between HS and acne vulgaris (AV), especially its more severe variant acne conglobata, has been noted due to high comorbidity rates of AV in HS patients compared to healthy controls (3, 4). Existing evidence supports a positive response shared by a proportion of patients diagnosed with either disease to treatment such as oral isotretinoin, 5-aminolevulinic acid photodynamic therapy, and, debatably, biologic therapies (5–10), suggesting possible mechanistic overlaps between HS and AV, in which pilosebaceous-apocrine unit component sebaceous glands (SGs) are considered to play a central role in disease development (11). Consequently, a critical unresolved question arises regarding how SGs might participate in the pathogenesis of HS.

The SG is an integral part of the pilosebaceous-apocrine unit and plays a critical role in maintaining cutaneous homeostasis through its lipid biosynthesis and immunomodulatory functions (12). Loss of SG and inflammation of sebaceous duct (SD) have been implicated as being involved in the pathogenesis of scarring alopecia (13). Alterations in sebum production, the end product of SG differentiation and the main function of SG, are proven to be related to several inflammatory dermatoses including atopic dermatosis and AV (14, 15). Previous own research by Lu et al. (2017) demonstrated disappearance and architectural destruction of SGs in scarring folliculitis HS patients (16), consistent with findings by Kamp et al. (2011), which documented SG depletion in HS epidermis compared to healthy controls (17). Nevertheless, whether SG aberrances constitute a primary pathogenic driver of early HS or merely reflect inflammatory sequelae remains unclear. Resolving this temporal discordance could provide novel insights into the pathogenesis of HS.

To clarify the role of SGs in HS, clinical specimens of unaffected non-lesional skin (NLS, *n* = 6) and early lesions (LS, *n* = 12) characterized by inflammatory nodules (Hurley Stage I) from HS patients, as well as age-/sex-matched healthy control (HC, *n* = 8) were used in this study to distinguish the pathological changes (**Supplementary Table 1**). Molecular alterations on the transcriptional level among SGs from NLS, LS, and HC were delineated with the single-cell sequencing (scRNA-seq) technique, followed by clinical validation with histological staining. Cultured human SZ95 sebocytes were then used to investigate HS-specific SG characteristics *in vitro*, with bulk RNA sequencing (bulk RNA-seq) profiling transcriptional dysregulation and liquid chromatography-tandem mass spectrometry-based lipidomic analysis quantifying metabolic alterations.

**Figure 1:**
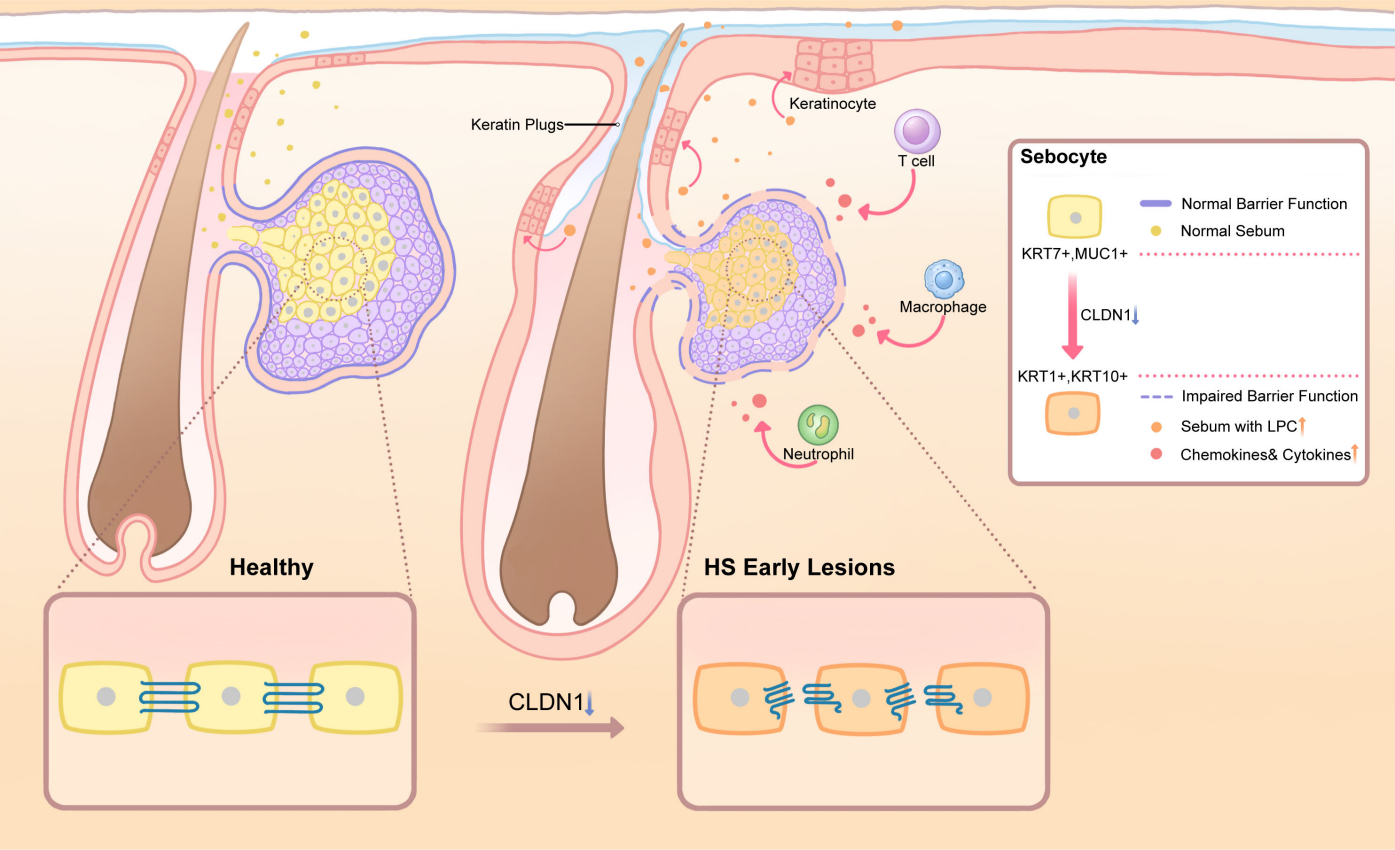
SGs in early Hidradenitis Suppurativa (HS) present size reduction, robust immunological engagement, and impaired barrier function characterized by loss of CLDN1, which induces inflammatory response activation, differentiation defects and metabolic reprogramming in sebocytes. Excessive lysophosphatidylcholine (LPC) in abnormally differentiated sebocytes leads to hyperproliferative and inflammatory phenotype in keratinocytes.

Our results suggested that SGs in HS early lesions presented differentiation abnormalities characterized by development issues and impaired cell junction networks, specifically loss of CLDN1, which would lead to disturbed sebocyte differentiation. Altered sebum production, in particular, the excessive secretion of lysophosphatidylcholine (LPC) in abnormally differentiated human SZ95 sebocytes, was associated with keratinocyte (KC) phenotypes in HS (**Figure 1**). We thereby proposed a primary involvement of SGs in early HS development through differentiation defects induced by CLDN1 downregulation and consequential lipid metabolism reprogramming. Not only did it complement the understanding of the pilosebaceous-apocrine unit’s pathogenic role in the initiation and aggravation of the disease, but it also implicated that the end product of SG differentiation, sebum, LPC in particular, could be a potential biomarker of disease activity and target for HS treatment, providing compelling evidence underscoring the need for expanded investigations into SG aberrations and SG-derived sebum signatures in HS pathogenesis.

## 2 Materials and methods

### 2.1 Participants and samples

Skin samples used for scRNA-seq and histological studies were surgically excised from NLS (skin with normal appearance > 5 cm away from any visible lesion in patients with HS), LS (newly formed, painful, inflamed papule or nodule), and healthy controls. Patients were diagnosed with sporadic HS with no clinically confirmed comorbidities, including metabolic or autoimmune disorders or concurrent dermatological conditions. Patients had no history of biologics, oral isotretinoin, surgical interventions within 6 months, systemic antibiotics within 3 months, or topical treatments within 1 month prior to sampling. Controls were screened to exclude inflammatory dermatoses and other health conditions. Each NLS sample and corresponding LS sample were taken from the same anatomical region in the same patient. This study was approved by the Ethics Committee of Renji Hospital, affiliated with Shanghai Jiaotong University School of Medicine (KY2021-113-B). Clinical information regarding skin samples for patients and healthy controls is detailed in **Supplementary Table 1**. Patients in this study have given written informed consent to publication of case details. No significant difference in age or sex between patients and controls was observed.

### 2.2 Histological study

#### 2.2.1 Sample preparation

Skin samples were fixed in formalin and embedded in paraffin before being cut into 50–100 vertical sections of 5 μm per specimen. Each of three sections from every 2 mm specimen was stained with hematoxylin & eosin.

#### 2.2.2 Immunohistochemical staining

All sections were deparaffinized, rehydrated, and washed before endogenous peroxidase was blocked using 3% hydrogen peroxide for 10 min. After water-bath heating for antigen retrieval, slides were incubated with primary antibodies β-catenin (1:500, GB11015, Servicebio, Wuhan, China), KRT16 (1:300, sc-53255, Santa Cruz, Shanghai, China), KRT10 (1:50, ab111447, Abcam, Hangzhou, China), KRT1 (1:1000, GB121018, Servicebio), and KRT7 (1:1000, GB115695, Servicebio), followed by horseradish peroxidase (HRP)–linked secondary antibodies and diaminobenzidine staining (ready-to-use #ZLI-9018, Zhong Shan Golden Bridge Biotechnology, Beijing, China). Counterstaining was done with hematoxylin. Slides were dehydrated with sequential ethanol washes (75%, 80, and 100%) for 1 min each.

#### 2.2.3 Immunofluorescence staining

The process was similar up to incubation with primary antibodies of CLDN1 (1:200, GB112543, Servicebio) and CD45 (1:1000, 13917, Cell Signaling Technology, Shanghai, China) at 4 °C overnight. Samples were incubated for 1h at room temperature after being washed with fluorescently labeled secondary antibodies, including HRP-labeled goat anti-rabbit (1:500, GB23303, Servicebio), CY3-labeled goat anti-rabbit (1:300, GB21303, Servicebio), and CY5-labeled goat anti-mouse (1:600, GB27301, Servicebio). Nuclei were counterstained with 4′,6-diamidino-2-phenylindole (DAPI, G1012, Servicebio). Sections were mounted using fluorescence mounting medium (G1401, Servicebio).

#### 2.2.4 SG size measurement

**Figure 2:**
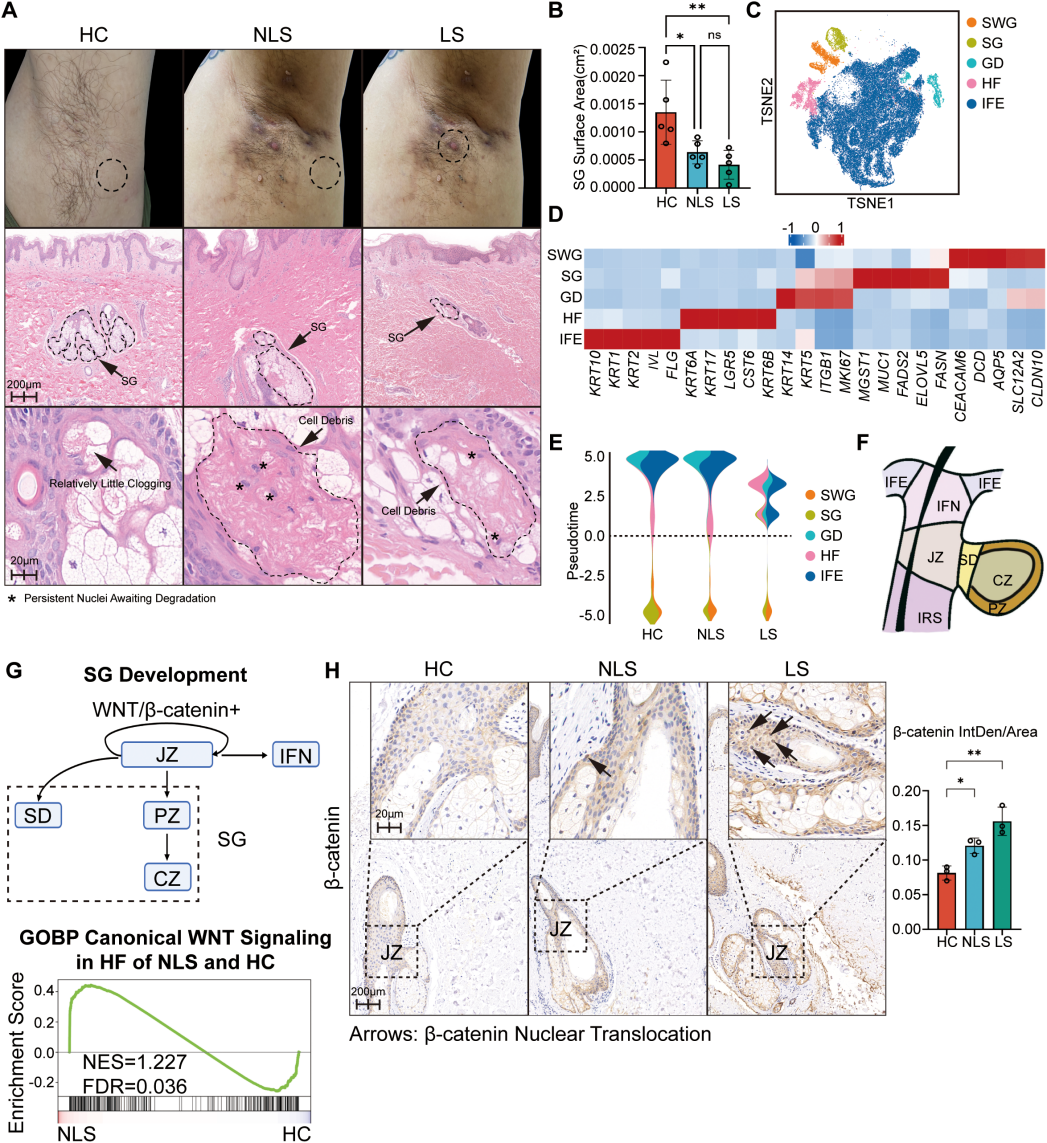
Incomplete holocrine secretion, size reduction, and development aberration in SGs from HS early lesions. **(A)** Representative pictures and hematoxylin& eosin staining of skin specimens of HC, NLS, and LS. Scale bar: 200 μm/20 μm for low/high magnification. **(B)** SG surface area per HF (*n* = 5). **(C)** TSNE visualization of epithelial cell in scRNA-seq. **(D)** Marker gene heatmap for cell clusters. Scale bar: relative expression. **(E)** Pseudo-time analysis of epithelial cells in scRNA-seq. **(F)** Upper pilosebaceous unit schematic. IFN, infundibulum; IRS, inner root sheath; PZ, peripheral zone; CZ, central zone. **(G)** WNT/β-catenin signaling in SG development schematic and GSEA enrichment of GOBP canonical WNT signaling (M12752) in scRNA-seq. **(H)** IHC staining and intensity quantification of β-catenin in HF-SG junctional zone (JZ) (*n* = 3). Scale bar: 200 μm/20 μm for low/high magnification. Bar graphs are presented with mean ± SD. *p < 0.05; **p < 0.01.

For SG size measurement, five patients (P2–P6) and five healthy donors (H1–H5) were analyzed. Each patient contributed one LS sample and one paired NLS sample. Each healthy donor contributed one HC sample. For each sample, the size of at least three SGs attached to different HF was measured. Quantitative analysis of SG size was performed by a light microscope (Olympus CX1, Tokyo, Japan) with a built-in camera (digital camera UCMOS05100KPA, Olympus). Using a ×20 objective lens, the surface area of SG was measured using software Caseviewer (3DHISTECH Ltd., version 2.4), outlining SG boundaries (excluding adjacent hair follicles and dermal tissues) and measuring size value in l μm^2^ (17). For each SG per HF, the mean area of three consecutive sections at the interval of 30–50 μm (determined by section sequence number) was calculated to compensate for tissue sectioning variability. The mean SG size per HF was calculated to represent a single data point per sample type per individual in **Figure 2B**.

#### 2.2.5 Semi-quantitative analysis of immunohistochemical and periodic acid–Schiff staining

**Figure 3:**
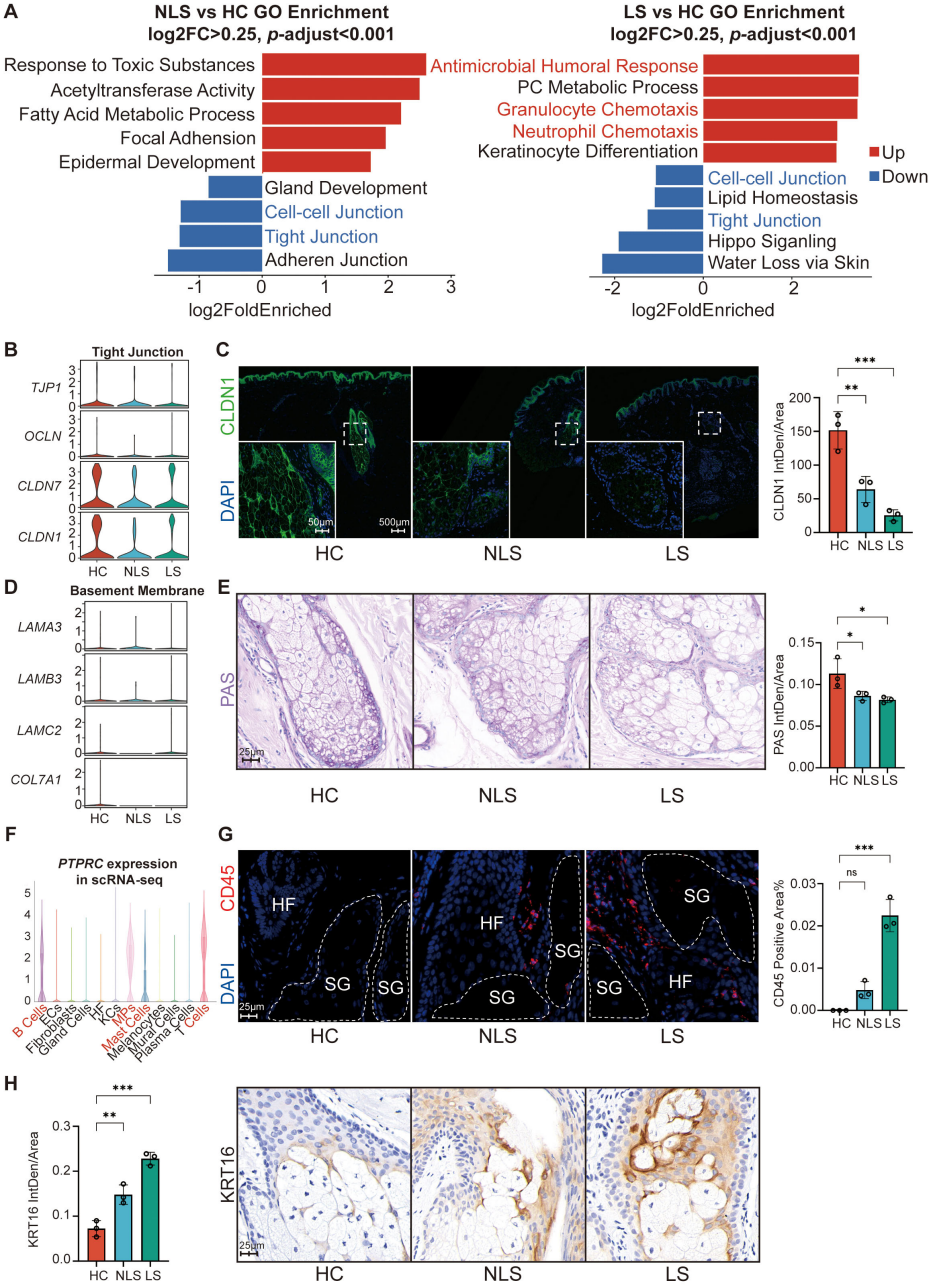
Impaired tight junction, compromised barrier integrity and robust immune engagement of SGs in HS early lesions. **(A)** Gene ontology enrichment of differential genes between SG cells in NLS and LS compared to HC. **(B)** Tight junction gene expression in scRNA-seq datasets. **(C)** IF staining and intensity quantification of CLDN1 in SGs (*n* = 3). Scale bar: 500μm/50μm for low/high magnification. **(D)** Basement membrane component gene expression in scRNA-seq datasets. **(E)** PAS staining and intensity quantification of SGs (*n* = 3). Scale bar: 25 μm. **(F)** *PTPRC* gene expression in cell clusters from scRNA-seq. **(G)** IF staining and CD45 positive area% (*n* = 3). Scale bar: 25 μm. **(H)** IHC staining and intensity quantification of KRT16 around SGs (*n* = 3). Scale bar: 25 μm. Bar graphs are presented with mean ± SD. ns: not significant, *p ≤ 0.05, **p < 0.01, ***p < 0.001.

Three patients from P3 to P12 and three healthy donors from H1 to H8 were included for each respective IHC and PAS analysis. Each patient contributed one LS sample and/or one NLS sample. Each healthy donor contributed one HC sample. Semi-quantitative analysis of IHC and PAS staining was performed using ImageJ software (National Institutes of Health, USA, version 1.54d), outlining boundaries for further measurement. DAB or PAS staining was separated from counterstain via H DAB or H PAS color deconvolution2. Threshold was set to isolate DAB or PAS-positive area (adjusting until brown or purple was distinct). Measurement of integrated density and area was taken to calculate mean gray value. For each sample, three sections were analyzed and the mean value was calculated to represent a single data point per sample type per individual in the final results in **Figures 2H**, **3E, H**, and **Figure 5G**.

**Figure 4:**
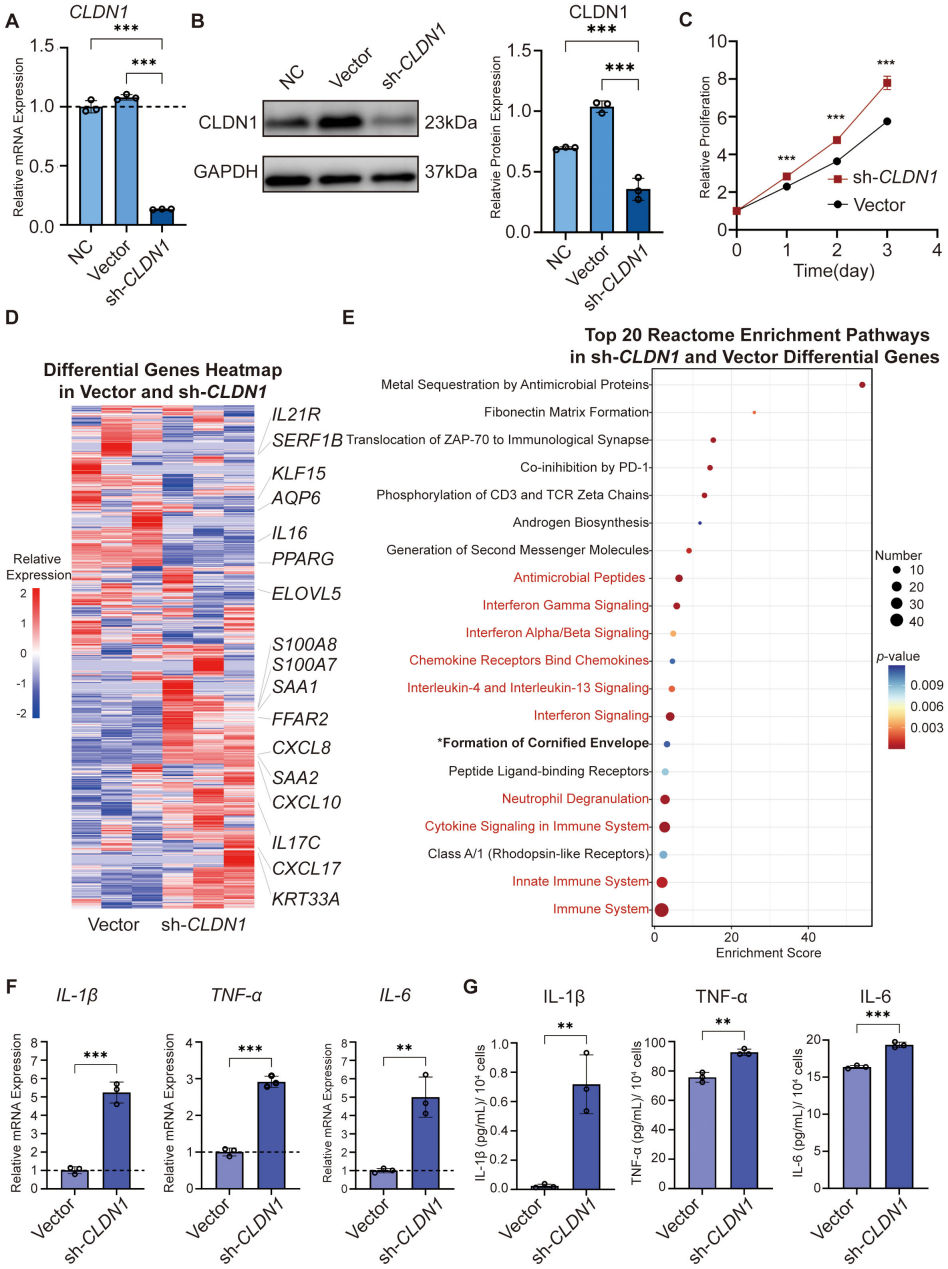
Loss of CLDN1 activates inflammatory response in human SZ95 sebocytes. **(A)** qPCR showing relative CLDN1 mRNA expression in Vector, NC and sh-*CLDN1*. **(B)** WB assay showing relative CLDN1 protein expression in Vector, NC and sh-*CLDN1*. **(C)** CCK8 assay showing relative proliferation rate of Vector and sh-*CLDN1* in 1–3 days. **(D)** Heatmap and representative differential genes of Vector and sh-*CLDN1* identified by bulk RNA-seq. **(E)** Top 20 differentially regulated pathways in sh-*CLDN1* compared to Vector. **(F)** qPCR showing IL-1β, TNF-α and IL-6 relative mRNA expression in Vector and sh-*CLDN1*. **(G)** ELISA showing concentration of IL-1β, TNF-α, and IL-6 in supernatant of Vector and sh-*CLDN1*. Bar graphs are presented with mean ± SD. **p < 0.01, ***p < 0.001.

#### 2.2.6 Semi-quantitative analysis of immunofluorescence staining

Three patients from P3 to P12 and three healthy donors from H1 to H8 were included for each respective IF analysis. Each patient contributed one LS sample and/or one NLS sample. Each healthy donor contributed one HC sample. Semi-quantitative analysis of IF staining was performed using ImageJ software, outlining boundaries for further measurement, calculating the mean gray value (**Figure 3C**) and the percentage of area with positive staining (**Figure 3G**). For each sample, three sections were analyzed and the mean value was calculated to represent a single data point per sample type per individual in the final results in **Figures 3C, G**.

### 2.3 Single-cell RNA sequencing

#### 2.3.1 Sample preparation

Fresh tissues were stored in the sCelLive™ Tissue Preservation Solution (Singleron, Nanjing, China) on ice after the surgery within 30 min. Specimens were washed with Hanks Balanced Salt Solution three times, minced into small pieces, and then digested with sCelLive™ Tissue Dissociation Solution (Singleron) by Singleron PythoN™ Tissue Dissociation System at 37°C for 15 min. Cell suspension was collected and filtered through a 40-micron sterile strainer. Afterwards, the GEXSCOPE^®^ red blood cell lysis buffer (RCLB, Singleron) was added, and the mixture [Cell: RCLB = 1:2 (size ratio)] was incubated at room temperature for 5–8 min to remove red blood cells. The mixture was then centrifuged at 300*g* at 4°C for 5 min to remove the supernatant and suspended softly with PBS (SH30256, Hyclone, Logan, USA). Finally, samples were stained with Trypan Blue and the cell viability was evaluated microscopically.

#### 2.3.2 Library preparation and analysis of single cell RNA sequencing data

Single cells were encapsulated into emulsion droplets by the Chromium Controller (10× Genomics). Libraries for scRNA-seq were built using the Chromium Single Cell 3' Library, Gel Bead & Multiplex Kit (10× Genomics, V2 and V3). Cells were loaded in each channel with a target output of ~5,000 cells. The cells were divided into gel beads in emulsion in the Chromium™ Controller instrument, where cell lysis and barcoded reverse transcription of RNA were processed. The cDNA was amplified with the Nextera XT DNA sample Pre-Kit (FC-131-1024, Illumina, San Diego, USA) to gain sequencing libraries. Libraries of individual samples were measured on the Agilent Bioanalyzer using a High Sensitivity DNA Kit ((Agilent) Technologies, USA), with the dataset uploaded to the National Genomics Data Center repository under the accession number HRA008409.

#### 2.3.3 Cell clustering and annotation

Clustering analysis was performed based on the “FindClusters” function of the Seurat package. The identified clusters were visualized on a two-dimensional map using the t-SNE method. To annotate the cell clusters, DEGs with high discrimination between groups were identified by the FindAllMarkers function in Seurat using the default non-parametric Wilcoxon rank sum test with Bonferroni correction. Cell groups were annotated based on DEGs and cellular markers acknowledged by previous literature.

### 2.4 Cell lines

Human SZ95 sebocytes were kindly gifted by professor Christos C. Zouboulis and his team from Brandenburg Medical School Theodor Fontane and Faculty of Health Sciences, Brandenburg, Dessau, Germany. HaCaT cells were purchased from the Cell Bank of the Chinese Academy of Sciences (catalog number: SCSP-5091), with cell line authentication performed by Genetic Testing Biotechnology on 2025.12.23 under the report number 251223. Cell lines used in this study have been tested and confirmed to be contamination free by microscopy and real-time quantitative polymerase chain reaction (qPCR).

All experiments in this study with cell lines involved were run in triplicate, except for lipidomic analysis in which six samples for each group were collected.

### 2.5 Cell culture

The immortalized human SG cell line SZ95 was maintained in Sebomed basal medium (Merck, Berlin, Germany), supplemented with 5 ng/ml human epidermal growth factor, 10% fetal bovine serum, 1% penicillin/streptomycin (all from Gibco, Invitrogen, Carlsbad, CA, USA), and 1 mM CaCl2 (Sigma-Aldrich, Shanghai, PR China) at 37°C in a humidified atmosphere containing 5% CO2. The immortalized HaCaT cell line was maintained in DMEM basal medium (Gibco, Melbourne, Australia), supplemented with 10% fetal bovine serum and 1% penicillin/streptomycin. The medium was replaced at intervals of every two days. Cells used in this study were between passages 30 and 40.

### 2.6 Knockdown of CLDN1

Lentivirus with CLDN1 targeting (5′-CTGGGAGTGATAGCAATCTTT-3′) and non-targeting shRNA control sequence was packaged, collected by OBIO Bio-Tech Inc. (Shanghai, China), and used to infect SZ95 cells at the same multiplicity of infection following the manufacturer’s instructions. Cells were selected after 48h of infection with 2 μg/ml puromycin before evaluation of CLDN1 mRNA and protein expression using qPCR and Western blot assay, respectively. Cells and cell lines were designated as NC (negative control with no lentivirus transfection), Vector (transfected with lentivirus carrying a control sequence), and sh-*CLDN1* (transfected with lentivirus carrying a CLDN1 targeting sequence), among which Vector and sh-*CLDN1* were to be maintained in complete medium with 0.5 μg/ml puromycin.

### 2.7 Quantitative polymerase chain reaction

**Figure 5:**
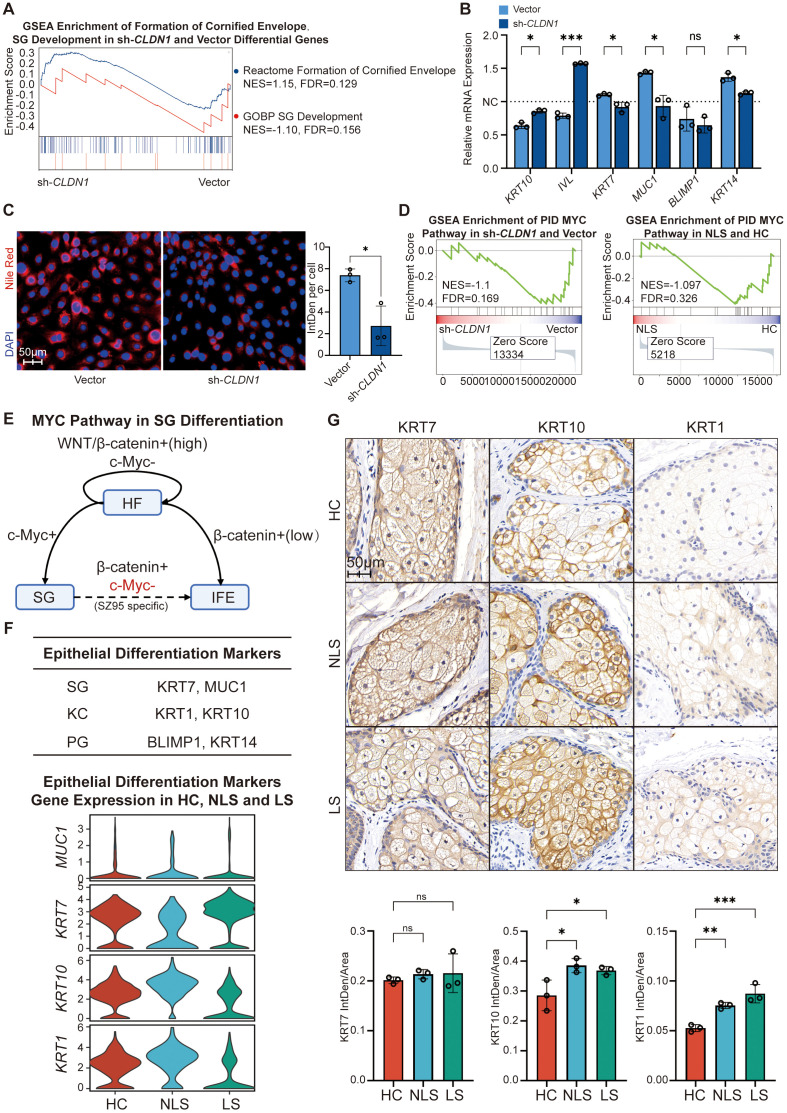
Abnormal sebocyte differentiation in sh-*CLDN1* and SGs from HS early lesions. **(A)** GSEA analysis of Reactome formation of cornified envelope (M27649) and SG development (M23880) pathway in bulk RNA-seq. **(B)** qPCR showing relative mRNA expression of epithelial differentiation markers. **(C)** Nile red staining and intensity quantification of Vector and sh-*CLDN1*. Scale bar: 50 μm (*n* = 3). **(D)** GSEA analysis of PID MYC pathway (M139) in bulk RNA-seq and scRNA-seq. **(E)** c-Myc regulation on SG differentiation schematic. **(F)** Differentiation markers for sebocytes, KC and PG (SG progenitor cells); expression of epithelial differentiation markers in SG cells from scRNA-seq. **(G)** IHC staining and intensity quantification of KRT7, KRT10 and KRT1 in SGs (*n* = 3). Scale bar: 50 μm. Bar graphs are presented with mean ± SD. *p < 0.05, **p < 0.01, ***p < 0.001.

Total RNA was extracted from cells upon reaching 80% confluency using an RNAeasy™ kit (R0024, Beyotime, Shanghai, China). RNA concentration was quantified by a NanoDrop spectrophotometer (Thermo Fisher Scientific, Shanghai, China) with an A260/A280 purity check within the range of 1.8–2.1 before reverse transcribing to cDNA using a PrimeScript™RT kit (RR036A, TaKaRa, Shiga, Japan). PCR experiments were run using 4 μl cDNA template per reaction and 0.5 μM forward & reverse primers with 5 μl 2× Universal Blue SYBR Green qPCR Master Mix (G3326, Servicebio) on a QuantStudio 7 Flex Real-Time PCR system. Nucleotide sequences of the primers are listed in **Supplementary Table 2**. Cycling conditions consisted of an initial 30 s of denaturation at 95 °C, followed by 40 cycles of 15 s of denaturation at 95 °C, 10 s of annealing at 60°C, and 30 s of elongation at 72°C. The expression level of target genes was normalized to GAPDH and target gene expression was calculated by the 2^-ΔΔCT^ method. Group NC (**Figure 4A**, **Figure 5B**) and Vector (**Figure 4F**) were used for further normalization, with results expressed in fold change.

### 2.8 Western blot

Seeded cells at a confluency of 80% were washed and lysed in cell lysis buffer (100 mM Tris-HCl, pH 6.8, 20% glycerol, 4% SDS, and 2% mercaptoethanol). Protein concentrations were determined using a BCA protein assay kit (Biotech Well, Shanghai, PR China). Equal amounts of protein were separated on SDS-PAGE and transferred to a nitrocellulose membrane. Blots were blocked in TBST (20 mM TrisHCl at pH 7.4, 150 mM NaCl, 0.1% Tween 20) containing 5% non-fat powder milk. The membranes were then incubated with CLDN1 (1:500, GB112543, Servicebio) and GAPDH (1:1000, 2118, Cell Signaling Technology) antibodies overnight at 4°C. The membranes were washed, incubated with secondary antibody (1:2000, 7074, Cell Signaling Technology) for 60 min at room temperature, and washed again. The blots were developed by the chemiluminescence system ECL (Amersham, Buckinghamshire, UK). Quantification was done using ImageJ, calculating the mean gray value of each blot. The level of target protein was normalized to GAPDH, with results expressed in fold change.

### 2.9 Cell Counting Kit 8 assay

Cells were seeded at 4000/well for 24h in a 96-well culture plate prior to assessment. On 0–3 days after seeding, the original medium was replaced by 100 μl of CCK8 solution (C0037, Beyotime) diluted to 1:10 in complete medium, respectively. The absorbance at 450 nm was measured after a 90-minute reaction using a multi-functional microplate reader (Biotek, Winooski, USA). The data of each group was normalized by the proliferation rate on day 0 to plot the cell proliferation curve.

### 2.10 Nile red staining

Cells were seeded at 4800/well for 24h in a 24-well culture plate and fixed with 4% PFA for 15 minutes before incubation with a 1:1000 diluted Nile Red and Hoechst 33342 solution using a commercial kit (C2051S, Beyotime) for 30 min. Samples were washed with PBS and examined using a ZOE™ fluorescent cell imager (Bio-rad, Hercules, USA). Semi-quantitative analysis was performed using ImageJ, outlining boundaries for further measurement and calculating mean gray intensity for each cell.

### 2.11 Immunocytochemistry

Cells were seeded in a 24-well culture plate for 24h to reach a confluency of 40%–50% before being fixed with 4% PFA for 15 min, permeabilized using 0.1% Triton X-100 (P0096, Beyotime) for 15 min, and blocked with 300 μl/well QuickBlock™ solution (P0220, Beyotime) for 15 min. Incubate with primary antibody targeting c-MYC (1:200, 80845-1-RR, Proteintech, Wuhan, China) at 4 °C overnight, followed by fluorescent secondary antibody (1:500, Thermo Fisher Scientific) for 1h at room temperature. Counterstain nuclei with Antifade Mounting Medium with DAPI (P0131, Beyotime) and image under ZOE™ fluorescent cell imager. All steps include PBS washes. Semi-quantitative analysis was performed using ImageJ, outlining boundaries for further measurement and calculating mean gray intensity for each cell.

### 2.12 Enzyme-linked immunosorbent assay

SZ95 cells were seeded in a six-well culture plate for 3 days to reach 90%–100% confluency, with 2 ml complete medium given to each well. HaCaT cells were seeded at 600000 cells/well in a six-well culture plate for 24h, washed, given 10μM LPC or vehicle (H_2_O) in 2 ml complete medium in each well, and cultured for another 24h. Supernatants were collected, centrifuged, and examined for target substance concentration levels within 2h using commercial ELISA kits for LPC (ZC-58155-J, ZCIBIO Technology, Suzhou, China), IL-1β (ES-H0341, BioQuanti, San Diego, USA), TNF-α (ES-H0853, BioQuanti), and IL-6 (ES-H0352, BioQuanti), respectively according to the manufacturer’s instructions, while cultured cells were digested with trypsin (25200056, Thermo Fisher Scientific) to perform cell counting using a cell counter (CellDrop, DeNovix, Portland, USA). The level of target substance concentration was normalized to cell number, with results expressed in substance concentration per 10^4^ cells.

### 2.13 Bulk RNA sequencing

Total RNA was extracted from sebocytes using Trizol (Invitrogen, Carlsbad, CA, USA) following the manufacturer’s instructions. RNA sequencing and the bioinformatic analysis were performed at OEbiotch, with the dataset uploaded to the Sequence Read Archive repository under the accession number PRJNA1394495.

### 2.14 Liquid chromatography-tandem mass spectrometry-based lipidomic analysis

Cultured cells were digested upon reaching 80% confluency with trypsin before centrifugation at 3000 rpm, 4 °C, for 5 min to remove supernatant. Pellet was resuspended in 4 °C PBS, transferred to fresh labeled tubes, and centrifuged at 3000 rpm, 4 °C for 5 min to remove supernatant two times before resuspension with 4 °C PBS. Cell counting was performed to ensure a sample size of more than 5E6 cells per sample before resuspension with 4 °C PBS and centrifugation at 3000 rpm, 4 °C, for 5 min to remove supernatant. The cell pellet was flash-freezed by immersing the tube bottom in liquid nitrogen for 1 min before brief pressure equalization to prevent tube cracking. Samples were sealed and stored at −80 °C until further processing. Liquid chromatography-tandem mass spectrometry-based lipidomic analysis was performed at OEbiotch, with the dataset uploaded to the Metabolights repository under the accession number MTBLS13578.

### 2.15 Statistical analysis

**Figure 6:**
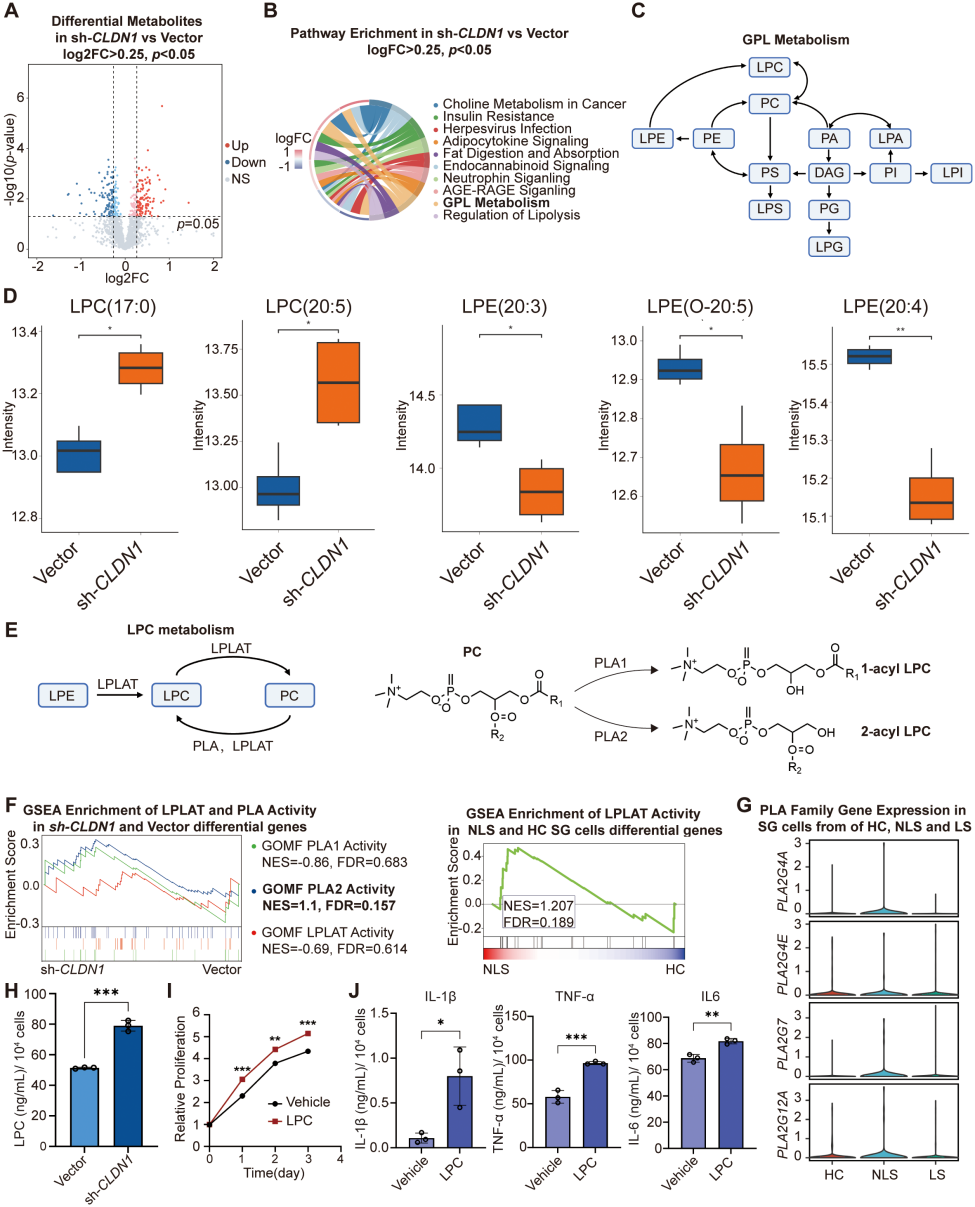
Excessive LPC production in sh-*CLDN1* induces HS phenotypes in KCs. **(A)** Differential metabolites volcano map in Vector and sh-*CLDN1*. **(B)** Reactome metabolic pathways enrichment in Vector and sh-*CLDN1*. **(C)** GPL metabolism schematic. **(D)** Metabolites intensity in sh-*CLDN1* and Vector. **(E)** LPC metabolism schematic. **(F)** GSEA analysis of LPLAT, PLA1, and PLA2 activity (M26325, M18817, M18390) in bulk RNA-seq and scRNA-seq. **(G)** PLA family gene expression in SG cells from scRNA-seq. **(H)** ELISA showing LPC concentration in Vector and sh-*CLDN1* supernatant. **(I)** CCK8 assay showing HaCaT proliferation rate treated with vehicle and LPC in 1–3 days. **(J)** ELISA showing concentration of IL-1β, TNF-α, and IL-6 in HaCaT supernatant treated with vehicle and LPC for 24h. Bar graphs are presented with mean ± SD. * *p* < 0.05, **p < 0.01, ***p < 0.001.

Statistical analysis was performed using Prism 10.0 (GraphPad Software, San Diego, CA, USA). One-way ANOVA with Šidák multiple comparison test (**Figures 2B, H**; **Figures 3C, E, G, H**; **Figures 4A, B**; **Figure 5G**), differentiation expression analysis (**Figures 3A, F**), two-tailed Student’s t-test (**Figure 4F, G**, **5C**, **6H, J**), multiple t-tests with false discovery rate correction (**Figure 4C**, **6I**) and Holm-Šidák multiple comparison tests (**Figure 5B**) were performed, with *p* < 0.05 defined as significant. |NES|>1.0, FDR <0.25 was defined as significant in GSEA analysis.

## 3 Results

### 3.1 Incomplete holocrine secretion, size reduction, and development aberration in SGs from HS early lesions

Hematoxylin & eosin staining was used to investigate the histopathological characteristics of SGs from patients and controls. In HC SGs, terminally differentiated sebocytes underwent holocrine secretion, a unique programmed cell death that involved nuclear DNA fragmentation, cell disintegration, and release of cellular contents through the SD into the HF (18). In NLS and LS SGs, a disruption of this process was observed, with incompletely degenerated sebocytes accumulating adjacent to the HF characterized by stacks of residual cell membrane debris (stained pink by eosin) and persistent nuclei (stained purple by hematoxylin) awaiting degradation (**Figure 2A**).

The size of SG from each sample was also measured, and the mean area of SG per HF from NLS was on average approximately 47.26% of those from HC (*p* = 0.04). The percentage dropped to 30.74% in LS compared to HC (*p* = 0.006) (**Figure 2B**).

To identify the reason for SG holocrine secretion disruption and size reduction, scRNA-seq was performed on samples obtained from HC, NLS, and LS (*n* = 3 each). A total of 133,046 cells were divided into 13 broad cell types using unsupervised clustering (**Supplementary Figure 1A**). Epithelial cells were further divided into five subgroups based on common lineage markers, naming interfollicular epidermis (IFE), HF, gland duct (GD), sweat gland (SWG), and SG (**Figure 2C**). SG was identified by marker genes *MGST1*, *MUC1*, *FADS2*, *ELOVL5*, and *FASN* (**Figure 2D**). Monocle pseudotime analysis showcased a much higher proportion of HF cells retaining around zero point, a relatively primitive phase on the differentiation axis, as opposed to the smaller number that proceeded to populate SGs in NLS and LS compared to HC (**Figure 2E**).

The upper part of HF known as the SG-HF junctional zone (JZ) provided progenitors for both SG and IFE, posing great significance to SG replenishment (**Figure 2F**). Activation of WNT/β-catenin signaling in JZ had been demonstrated to cause cell proliferation in HF and SG atrophy (19, 20). Gene set enrichment analysis (GSEA) revealed a significant upregulation of WNT/β-catenin signaling in HF from NLS compared to HC (NES = 1.227, FDR = 0.036) (**Figure 2G**). Immunohistochemical (IHC) staining confirmed that expression of β-catenin was significantly higher in HF from both NLS and LS compared to HC (*p =* 0.03, *p* = 0.002), with nuclear translocation observed in JZ from LS, symbolizing activation of WNT/β-catenin signaling (**Figure 2H**). These findings suggested that SG size reduction in HS early lesions might be associated with abnormal stem cell activities in the HF, while it still remained unclear what had caused the incomplete holocrine secretion in terminally differentiated sebocytes.

### 3.2 Impaired tight junction, compromised barrier integrity, and robust immune engagement of SGs in HS early lesions

Gene Ontology pathway enrichment analysis summarized dysregulation of several cellular and metabolic processes, with cell–cell junction being one of the most downregulated pathways in both NLS and LS SG cells compared to HC. Among the five types of cell–cell junctions, tight junction signaling was notably suppressed in NLS and LS SG cells compared to HC. In addition, a series of antimicrobial immune reaction-related pathways, including antimicrobial humoral response, granulocyte migration, and neutrophil chemotaxis, were found to be upregulated in LS compared to HC (**Figure 3A**).

To further validate the above findings, specific markers for cell-cell junction and immune response were selected and monitored on both transcriptional and protein levels. Gene expression of tight junction components TJP1, OCLN, CLDN7, and CLDN1 crucial for maintaining tight junction integrity showed a unified decrease in NLS compared to HC (**Figure 3B**), with *p*-value for each paired comparison presented in **Supplementary Table 3**. IF staining confirmed a significant downregulation of CLDN1 in SGs of NLS and LS compared to HC (*p =* 0.004, *p* < 0.001) (**Figure 3C**). Cell junctions and basement membrane worked together as a dual-layered system for tissue barrier integrity (21, 22). Expression of key basement membrane component genes *LAMA3*, *LAMB3*, *LAMC2*, and *COL7A1* was found to be dysregulated in SG cells from HS patients compared to HC (**Figure 3D**). PAS staining showcased a strong and consistent pattern in SGs in HC, while the intensity of staining decreased significantly in NLS and LS SGs (*p =* 0.05, *p* = 0.03) (**Figure 3E**).

A combination of CD45 and KRT16 were chosen as biomarkers for immune response due to their acknowledged application in evaluation of inflammatory dermatosis activity (23, 24). Cells expressing the PTPRC gene, which encoded CD45 were found to be mainly expressed in immune cells such as mononuclear phagocytes (MPs), T cells, mast cells, and melanocytes (**Figure 3F**). IF staining showed a small number of CD45^+^ cells in area adjacent to SGs in NLS and the pool significantly grew in size to infiltrate similar regions in LS, while few CD45^+^ cells were found around SGs in HC (*p* = 0.11, *p* < 0.001) (**Figure 3G**). Skin barrier alarmin KRT16 was identified by IHC staining to significantly increase in NLS and LS compared to HC (*p* = 0.004, *p* < 0.001) (**Figure 3H**).

Taken together, these results suggested that the barrier integrity of SGs was compromised in early lesions of HS. Considering that the tissue barrier served as the first line of defense in the immune system, it was possible that the loss of barrier function might be the bridge connecting SG aberration to HS pathogenesis, especially regarding SG’s contribution to early inflammatory infiltration found in this study.

### 3.3 Loss of *CLDN1* activates inflammatory response in human SZ95 sebocytes

To unravel how the impaired tight junction in SGs participated in HS pathogenesis, lentivirus was used to knock down the *CLDN1* gene in human SZ95 sebocytes (25). qPCR demonstrated that the expression of *CLDN1* on the transcriptional level in sh-*CLDN1* was significantly downregulated to below 30% of that in Vector and NC (**Figure 4A**). The Western blot assay confirmed that the protein level of CLDN1 was also significantly suppressed in sh-*CLDN1* compared to Vector and NC (**Figure 4B**). The CCK8 assay demonstrated that loss of CLDN1 significantly promoted proliferation in human SZ95 sebocytes in 1–3 days (**Figure 4C**). Bulk RNA-seq was then used to delineate the global gene expression of sh-*CLDN1* and Vector (*n* = 3). Among the 243 differential genes, 142 genes were significantly upregulated in sh-*CLDN1*, including fatty acid synthesis gene *FFAR2* and inflammatory mediator genes *SAA1*, *S100A8*, *S100A7*, *CXCL8*, *IL17C*, *KRT33A*, while 101 genes were significantly downregulated, including cell junction component gene *AQP6* and lipid synthesis gene *KLF15*, *PPARG* (**Figure 4D**). Pathway enrichment analysis revealed several enhanced signaling pathways related to inflammation and immune response, including antimicrobial peptides, interferon gamma signaling, and cytokine signaling in the immune system (**Figure 4E**). qPCR verified that the expression of *IL-1*β, *TNF-*α, and *IL-6* was significantly upregulated on the transcriptional level in sh-*CLDN1* compared to Vector (*p* < 0.001, *p* < 0.001, *p* = 0.003) (**Figure 4F**), with significantly elevated level of these cytokines detected by ELISA in sh-*CLDN1* supernatant (*p* = 0.004, *p* < 0.001, *p* < 0.001) (**Figure 4G**).

### 3.4 Abnormal sebocyte differentiation in sh-*CLDN1* and SGs from HS early lesions

In addition to the increased inflammatory activities, the pathway of cornified envelope formation was also found to be upregulated in sh-*CLDN1* (**Figure 4E**), which was curious given that sebocytes, unlike KCs, did not usually participate in the formation of cornified envelope. GSEA enrichment further identified a significant upregulation of the formation of cornified envelope pathway along with downregulation of the SG development pathway in sh-*CLDN1* compared to Vector (NES = 1.15, FDR = 0.129; NES = −1.1, FDR = 0.156) (**Figure 5A**). Markers for epithelial cell differentiation were then assessed by qPCR, and sh-*CLDN1* presented a significant upregulated mRNA expression of mature KC markers *KRT1*, *IVL*, and downregulation of mature sebocyte markers *MUC1*, *KRT7* compared to Vector (*p* = 0.01, *p* < 0.001, *p* = 0.03, *p* = 0.02) (**Figure 5B**). Nile red staining revealed a significant decrease in general sebum production in sh-*CLDN1* compared to Vector, indicating abnormal holocrine secretion in sh-*CLDN1* (*p* = 0.01) (**Figure 5C**).

Upon examining key pathways in regulating sebocytes differentiation, GSEA analysis revealed that the c-Myc pathway, a key lineage-determining signaling in SZ95 sebocytes (**Figure 5E**), was significantly suppressed in sh-*CLDN1* (NES = −1.1, FDR = 0.169). Immunocytochemistry assay further confirmed a significant decrease in total Myc protein expression (*p* = 0.003) (**Supplementary Figure 2**). This suppression was also noted in SG cells from NLS when compared to HC according to scRNA-seq (NES = −1.097, FDR = 0.326), prompting us to explore whether the abnormal differentiation of sebocytes was also present in SGs from early HS lesions (**Figure 5D**). While gene ontology enrichment had already identified downregulation of gland development and upregulation of keratinization pathway in both NLS and LS SGs compared to HC (**Figure 3A**), GSEA enrichment further confirmed a significant upregulation of keratinization pathway in SGs from NLS and LS compared to HC (**Supplementary Figures 1B, C**). Decreased SG marker genes *KRT7*, *MUC1*, and increased KC marker genes *KRT1*, *KRT10* were also found in SG cells from NLS and LS by scRNA-seq, with *p*-value for each paired comparison presented in **Supplementary Table 4** (**Figure 5F**). While the alteration of KRT7 expression was insignificant (*p* = 0.8, *p* = 0.74), IHC staining revealed a significantly elevated level of KRT10 and KRT1 in SGs from NLS and LS compared to HC (*p* = 0.02, *p* = 0.05; *p* = 0.007, *p* < 0.001) (**Figure 5G**). These findings, along with the incomplete holocrine secretion observed in NLS and LS SGs, supported a differentiation defect of sebocytes in early HS, while its association with disease pathogenesis remained unknown.

### 3.5 Excessive LPC production in sh-*CLDN1* induces HS phenotypes in keratinocytes

We proceeded to investigate the characteristics of abnormally differentiated sebocytes from the perspective of sebum, the end product of sebocyte differentiation and main content of holocrine secretion (26). Metabolites produced by sh-*CLDN1* and Vector were distinguished by liquid chromatography-tandem mass spectrometry-based lipidomic analysis (*n* = 6). A total of 109 metabolites were found to be significantly downregulated, while 119 metabolites were significantly upregulated in sh-*CLDN1* (**Figure 6A**). Glycerophospholipid (GPL) metabolism pathway was among one of the most upregulated pathways in sh-*CLDN1* compared to Vector (*p* < 0.001) (**Figure 6B**). Among the elements of GPL metabolism (**Figure 6C**), LPC (17:0) and LPC (20:5) were significantly upregulated, while lysophosphatidylethanolamine (LPE) (20:3), LPE (O-20:5), LPE (20:4) were significantly downregulated in sh-*CLDN1* compared to Vector (**Figure 6D**). LPC (17:0) and LPC (20:5) were both subclasses of LPC, a minor component in sebum most regularly observed to be elevated in skin disorders such as psoriasis and rosacea (27, 28).

As shown in **Figure 6E**, LPC metabolism relied heavily on phosphatidylcholine acylhydrolase (PLA) and lysophospholipid O - acyltransferase (LPLAT) family enzymes, with the two types of PLAs converting phosphatidylcholine (PC) into LPC and participation of LPLAT family enzymes in LPC production/remodeling (29). GSEA analysis revealed a significant increase of PLA2 activity in sh-*CLDN1* compared to Vector (NES = 1.1, FDR = 0.157) and of LPLAT activity in NLS compared to HC (NES = 1.207, FDR = 0.1885), suggesting a possible upregulation of LPC production in HS early lesions (**Figure 6F**). Gene expression of PLA2 family enzymes *PLA2G4A*, *PLA2G4E*, *PLA2G7*, and *PLA2G12A* was also found to be upregulated in NLS compared to HC (**Figure 6G**). ELISA confirmed that LPC was significantly increased in sh-*CLDN1* supernatant (**Figure 6H**). One of the most acknowledged players in HS, KCs, were then treated with LPC, and the CCK8 assay demonstrated that LPC induced proliferation of HaCaT KCs in 1–3 days (*p* < 0.001, *p* = 0.001, *p* < 0.001) (**Figure 6I**). Significant elevation of inflammatory mediators IL-1β, TNF-α, and IL-6 was detected by ELISA in supernatants of HaCaT cells treated with LPC for 24h (*p* = 0.02, *p* < 0.001, *p* = 0.003) (**Figure 6J**), suggesting that exogenous LPC was able to induce HS phenotypes in KCs.

## 4 Discussion

In this study, we provide evidence for SG differentiation defects as a primary instigator rather than a consequence of inflammation in HS.

First, we demonstrate that SG size reduction in HS patients constitutes an early pathogenic event, with possible association to aberrant lineage commitment of SG stem cells within the HF. Accumulation of JZ stem cells in HF at the expense of SG is in favor of HS development, as these cells also contribute to the infundibulum (30) and occasionally IFE lineage (19), potentially participating in the early follicular hyperkeratosis of HS.

Second, our work reveals tight coupling between barrier disruption and innate immune activation in early HS SGs. Previous evidence has already suggested that SGs were prone to rupture and release inflammatory mediators when exposed to mechanical stress in HS patients (31). Building upon our prior discovery of cell junction dysregulation, which destabilizes apocrine glands in HS patients (32), we now identify a parallel breakdown in SGs characterized by downregulation of key tight junction molecules, including CLDN1, TJP1, OCLN, and CDH1. Although TJ alterations have been implied as early events in atopic dermatosis and psoriasis (33–35), the spatial dynamics of such defects in HS seem gland-specific, since previous studies reveal no difference in epidermis TJ components in HS early development (36). Specifically, downregulation of CLDN1 not only compromises SG barrier integrity, but it also directly triggers a robust pro-inflammatory response in sebocytes *in vitro*, transforming them into inflammatory amplifiers. Loss of CLDN1 in sebocytes induces increased gene expression of chemokines (e.g., *CXCL8*, *CXCL10*) and overproduction of proinflammatory cytokines, recruiting and activating CD45^+^ immune cells to establish a chronic inflammatory microenvironment conducive to HS progression.

In addition to orchestrating inflammatory responses, CLDN1 has been proved to be essential for the terminal differentiation of sebocytes in mice, as loss of CLDN1 disrupts proper holocrine secretion via incomplete sebocyte degradation and subsequent ductal obstruction (37). In this study, not only do we observe an accumulation of incompletely degenerated sebocytes in SD of NLS and LS SGs, but a KC-like phenotype is also noted *in vitro* with *CLDN1*-knockdown human SZ95 sebocytes accompanied by c-Myc suppression. SZ95 cells have been demonstrated to harbor the capacity of differentiating either along the sebaceous lineage with c-Myc activation or toward KCs by c-Myc downregulation (38). Our previous study demonstrated that *P. acnes* was able to induce a similar KC lineage de-differentiation associated with comedonegenesis in AV in SZ95 cells (39). Critically, this lineage reprogramming by *CLDN1* knockdown corresponds with the epithelial differentiation marker profile in SGs from HS early lesions. Coupled with incomplete holocrine secretion, these findings support that sebocyte differentiation defect is a primary event during early disease pathogenesis.

Finally, we extend our research to sebum, the end product of sebocyte differentiation. Our study provides first evidence for a novel mechanistic hypothesis linking SG dysfunction to broader skin pathological changes. Metabolic reprogramming caused by CLDN1 loss in sebocytes, especially the accumulation of bioactive lipid LPC, promotes proliferation and inflammation in KCs, exacerbating HS hallmark HF hyperkeratosis and infundibulum occlusion, leading to recurrent, painful nodules and abscesses. An elevated level of LPC has been documented in rosacea lesions (28) and was proved to participate in the pathogenesis of psoriasis by inducing the production of IL-1β in KCs, which consequently facilitated T-cell differentiation (27).

Limitations of this study lie in a primary reliance on the SZ95 cell line, which is divergent from human SG cells, an undefined upstream mechanism for CLDN1 downregulation in SGs from HS early lesions, and a lack of clinical evidence for LPC-induced HS phenotype hypothesis. In addition, the generalizability of our key pathologic findings, such as early SG atrophy coupled with aberrant stem cell activity to all HS phenotypes requires cautious interpretation due to an underrepresentation of high-exposure patients (e.g., genetic variation, smoking, and obesity) and the absence of data correlating treatment efficacy with pathologic endotypes. Further research will prioritize dissecting SG traits across risk-stratified patient subpopulations as well as divergent therapeutic responders, elucidating regulators of CLDN1, clinical validation of SG metabolic reprogramming in HS pathogenesis, and development of LPC-targeted interventions for early HS.

## Data Availability

The datasets presented in this study can be found in online repositories. The names of the repository/repositories and accession number(s) can be found in the article/**Supplementary Material**.

## References

[1] Zouboulis CC Benhadou F Byrd AS Chandran NS Giamarellos-Bourboulis EJ Fabbrocini G . What causes hidradenitis suppurativa?-15 years after. Exp Dermatol. (2020) 29:1154–70. doi: 10.1111/exd.14214 33058306

[2] Zouboulis CC Nogueira da Costa A Makrantonaki E Hou XX Almansouri D Dudley JT . Alterations in innate immunity and epithelial cell differentiation are the molecular pillars of hidradenitis suppurativa. J Eur Acad Dermatol Venereol. (2020) 34:846–61. doi: 10.1111/jdv.16147 31838778

[3] Phan K Charlton O Smith S . Hidradenitis suppurativa and acne vulgaris and conglobata—systematic review and meta-analysis. Biomed Dermatol. (2019) 3:12. doi: 10.1186/s41702-019-0045-z 38164791

[4] Wertenteil S Strunk A Garg A . Overall and subgroup prevalence of acne vulgaris among patients with hidradenitis suppurativa. J Am Acad Dermatol. (2019) 80:1308–13. doi: 10.1016/j.jaad.2018.09.040 30287328

[5] Gold M Bridges TM Bradshaw VL Boring M . Ala-pdt and blue light therapy for hidradenitis suppurativa. J Drugs Dermatol. (2004) 3:S32–5. 14964759

[6] Hongcharu W Taylor CR Chang Y Aghassi D Suthamjariya K Anderson RR . Topical ala-photodynamic therapy for the treatment of acne vulgaris. J Invest Dermatol. (2000) 115:183–92. doi: 10.1046/j.1523-1747.2000.00046.x 10951234

[7] Kemény L Degovics D Szabó K . Is there a place for biologics in acne? Am J Clin Dermatol. (2025) 26:667–76. doi: 10.1007/s40257-025-00954-8 40474034 PMC12436545

[8] Patel N McKenzie SA Harview CL Truong AK Shi VY Chen L . Isotretinoin in the treatment of hidradenitis suppurativa: a retrospective study. J Dermatol Treat. (2021) 32:473–5. doi: 10.1080/09546634.2019.1670779 31535587

[9] Ping Y Jian Bo Z Xing Yun Z Ali K Jun C Xu Lou I . Case report: Acne vulgaris treatment with 5-aminolaevulinic acid photodynamic therapy and adalimumab: a novel approach. Front Med (Lausanne). (2023) 10:1187186. doi: 10.3389/fmed.2023.1187186 37250640 PMC10213406

[10] Wang P Wang B Zhang L Liu X Shi L Kang X . Clinical practice guidelines for 5-aminolevulinic acid photodynamic therapy for acne vulgaris in China. Photodiagnosis Photodyn Ther. (2023) 41:103261. doi: 10.1016/j.pdpdt.2022.103261 36587863

[11] Zouboulis CC . Acne and sebaceous gland function. Clin Dermatol. (2004) 22:360–6. doi: 10.1016/j.clindermatol.2004.03.004 15556719

[12] Zouboulis CC Coenye T He L Kabashima K Kobayashi T Niemann C . Sebaceous immunobiology - skin homeostasis, pathophysiology, coordination of innate immunity and inflammatory response and disease associations. Front Immunol. (2022) 13:1029818. doi: 10.3389/fimmu.2022.1029818 36439142 PMC9686445

[13] Al-Zaid T Vanderweil S Zembowicz A Lyle S . Sebaceous gland loss and inflammation in scarring alopecia: a potential role in pathogenesis. J Am Acad Dermatol. (2011) 65:597–603. doi: 10.1016/j.jaad.2010.09.774 21669475

[14] Clayton RW Göbel K Niessen CM Paus R van Steensel MAM Lim X . Homeostasis of the sebaceous gland and mechanisms of acne pathogenesis. Br J Dermatol. (2019) 181:677–90. doi: 10.1111/bjd.17981 31056753

[15] Shi VY Leo M Hassoun L Chahal DS Maibach HI Sivamani RK . Role of sebaceous glands in inflammatory dermatoses. J Am Acad Dermatol. (2015) 73:856–63. doi: 10.1016/j.jaad.2015.08.015 26386632

[16] Lu L Lai H Pan Z Hu T Hou X Cao K . Clinical and histopathological characteristics in patients with scarring folliculitis type of acne inversa. Dermatoendocrinol. (2017) 9:e1361575. doi: 10.1080/19381980.2017.1361575 29484097 PMC5821155

[17] Kamp S Fiehn AM Stenderup K Rosada C Pakkenberg B Kemp K . Hidradenitis suppurativa: a disease of the absent sebaceous gland? Sebaceous gland number and volume are significantly reduced in uninvolved hair follicles from patients with hidradenitis suppurativa. Br J Dermatol. (2011) 164:1017–22. doi: 10.1111/j.1365-2133.2011.10224.x 21250966

[18] Schneider MR Paus R . Sebocytes, multifaceted epithelial cells: lipid production and holocrine secretion. Int J Biochem Cell Biol. (2010) 42:181–5. doi: 10.1016/j.biocel.2009.11.017 19944183

[19] Jensen KB Collins CA Nascimento E Tan DW Frye M Itami S . Lrig1 expression defines a distinct multipotent stem cell population in mammalian epidermis. Cell Stem Cell. (2009) 4:427–39. doi: 10.1016/j.stem.2009.04.014 19427292 PMC2698066

[20] Shang W Tan AYQ van Steensel MAM Lim X . Aberrant wnt signaling induces comedo-like changes in the murine upper hair follicle. J Invest Dermatol. (2022) 142:2603–2612.e6. doi: 10.1016/j.jid.2021.11.034 34929175

[21] Adil MS Narayanan SP Somanath PR . Cell-cell junctions: structure and regulation in physiology and pathology. Tissue Barriers. (2021) 9:1848212. doi: 10.1080/21688370.2020.1848212 33300427 PMC7849786

[22] Schwarzbauer J . Basement membranes: putting up the barriers. Curr Biology: CB. (1999) 9:R242–4. doi: 10.1016/s0960-9822(99)80153-5 10209113

[23] Cohen E Johnson CN Wasikowski R Billi AC Tsoi LC Kahlenberg JM . Significance of stress keratin expression in normal and diseased epithelia. iScience. (2024) 27:108805. doi: 10.1016/j.isci.2024.108805 38299111 PMC10828818

[24] Sidler D Wu P Herro R Claus M Wolf D Kawakami Y . Tweak mediates inflammation in experimental atopic dermatitis and psoriasis. Nat Commun. (2017) 8:15395. doi: 10.1038/ncomms15395 28530223 PMC5493595

[25] Zouboulis CC Seltmann H Neitzel H Orfanos CE . Establishment and characterization of an immortalized human sebaceous gland cell line (Sz95). J Invest Dermatol. (1999) 113:1011–20. doi: 10.1046/j.1523-1747.1999.00771.x 10594745

[26] Picardo M Ottaviani M Camera E MastroFrancesco A . Sebaceous gland lipids. Dermatoendocrinol. (2009) 1:68–71. doi: 10.4161/derm.1.2.8472 20224686 PMC2835893

[27] Liu P Zhou Y Chen C Yan B Li L Zhu W . Lysophosphatidylcholine facilitates the pathogenesis of psoriasis through activating keratinocytes and t cells differentiation via glycolysis. J Eur Acad Dermatol Venereol. (2023) 37:1344–60. doi: 10.1111/jdv.19088 37013729

[28] Yang Y Zhao Z Lu L Gao N Hu J Zhang X . Lipidomic profiling of skin surface lipids in a cohort of Chinese patients with rosacea. Sci Rep. (2025) 15:39163. doi: 10.1038/s41598-025-24539-x 41203722 PMC12594949

[29] Schagen S Perchuc A-M Voegeli R Imfeld D Schreier T Zouboulis CC . Lipid regulation in Sz95 sebocytes by active and inactive phospholipases A2 from Bothrops moojeni venom. Dermatoendocrinol. (2009) 1:102–7. doi: 10.4161/derm.1.2.7820

[30] Düz T Torocsik D Simmering A Wolf P Gallinat S Baumbach J . High-resolution spatial map of the human facial sebaceous gland reveals marker genes and decodes sebocyte differentiation. J Invest Dermatol. (2026) 146:40–54.e14. doi: 10.1016/j.jid.2025.04.041 40449655

[31] Danby FW Jemec GB Marsch W von Laffert M . Preliminary findings suggest hidradenitis suppurativa may be due to defective follicular support. Br J Dermatol. (2013) 168:1034–9. doi: 10.1111/bjd.12233 23320858

[32] Li J Li S Zhang Q Liang M Chen X Feng Y . Apocrine gland damage and the release of specific keratins in early stage indicate the crucial involvement of apocrine glands in hidradenitis suppurativa. J Invest Dermatol. (2025) 145:1371–1384.e7. doi: 10.1016/j.jid.2024.09.021 39547394

[33] Bäsler K Brandner JM . Tight junctions in skin inflammation. Pflugers Arch. (2017) 469:3–14. doi: 10.1007/s00424-016-1903-9 27853878

[34] Katsarou S Makris M Vakirlis E Gregoriou S . The role of tight junctions in atopic dermatitis: a systematic review. J Clin Med. (2023) 12(4):1538. doi: 10.3390/jcm12041538 36836073 PMC9967084

[35] Kirschner N Poetzl C von den Driesch P Wladykowski E Moll I Behne MJ . Alteration of tight junction proteins is an early event in psoriasis: putative involvement of proinflammatory cytokines. Am J Pathol. (2009) 175:1095–106. doi: 10.2353/ajpath.2009.080973 19661441 PMC2731128

[36] Somogyi O Dajnoki Z Szabó L Gáspár K Hendrik Z Zouboulis CC . New data on the features of skin barrier in hidradenitis suppurativa. Biomedicines. (2023) 11(1):127. doi: 10.3390/biomedicines11010127 36672635 PMC9855647

[37] Atsugi T Yokouchi M Hirano T Hirabayashi A Nagai T Ohyama M . Holocrine secretion occurs outside the tight junction barrier in multicellular glands: lessons from claudin-1-deficient mice. J Invest Dermatol. (2020) 140:298–308.e5. doi: 10.1016/j.jid.2019.06.150 31445004

[38] Lo Celso C Berta MA Braun KM Frye M Lyle S Zouboulis CC . Characterization of bipotential epidermal progenitors derived from human sebaceous gland: contrasting roles of c-myc and beta-catenin. Stem Cells. (2008) 26:1241–52. doi: 10.1634/stemcells.2007-0651 18308950

[39] Cao K Chen G Chen W Hou X Hu T Lu L . Formalin-killed propionibacterium acnes activates the aryl hydrocarbon receptor and modifies differentiation of Sz95 sebocytes *in vitro*. Eur J Dermatol. (2021) 31:32–40. doi: 10.1684/ejd.2021.3964 33648912

