# Supplementary material for: Differentiation defects reposition sebaceous glands as inflammatory instigators in the early pathogenesis of hidradenitis suppurativa
Source: Front Immunol. 2026 Jun 8;17:1785747. doi: 10.3389/fimmu.2026.1785747 (PMC13283874; doi:10.3389/fimmu.2026.1785747)
Supplement: Supplementary file 1 [file DataSheet1.docx]

Supplementary Material

# Supplementary Figures and Tables

- 1. **Supplementary Figures**


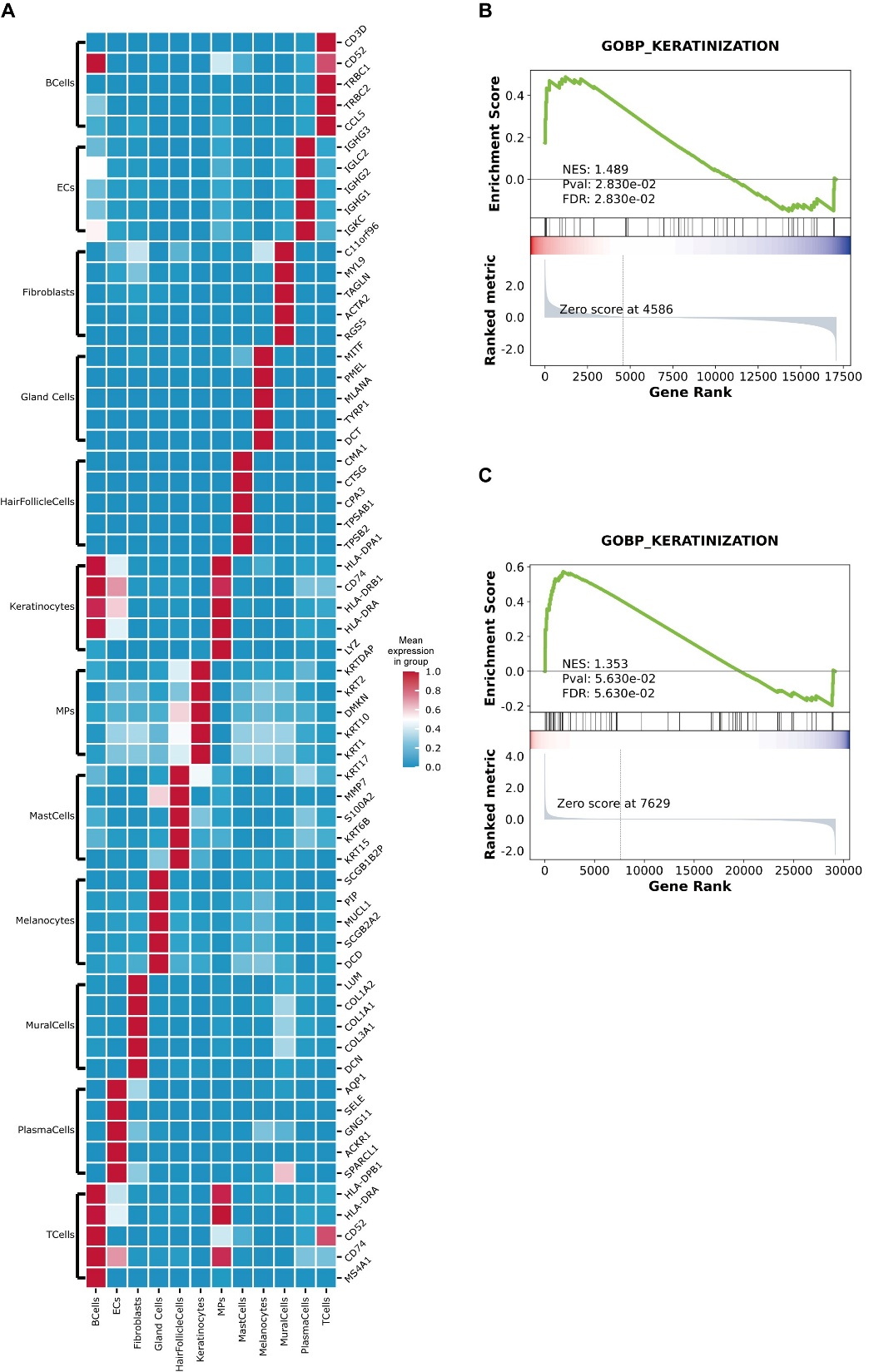


**Supplementary Figure 1.** Cell clusters and GSEA analysis in scRNA-seq dataset. (A) Cell clusters and marker genes in scRNA-seq dataset. (B) GSEA enrichment of GOBP keratinization pathway in SG cells from NLS compared to HC. (C) GSEA enrichment of GOBP keratinization pathway in SG cells from LS compared to HC.


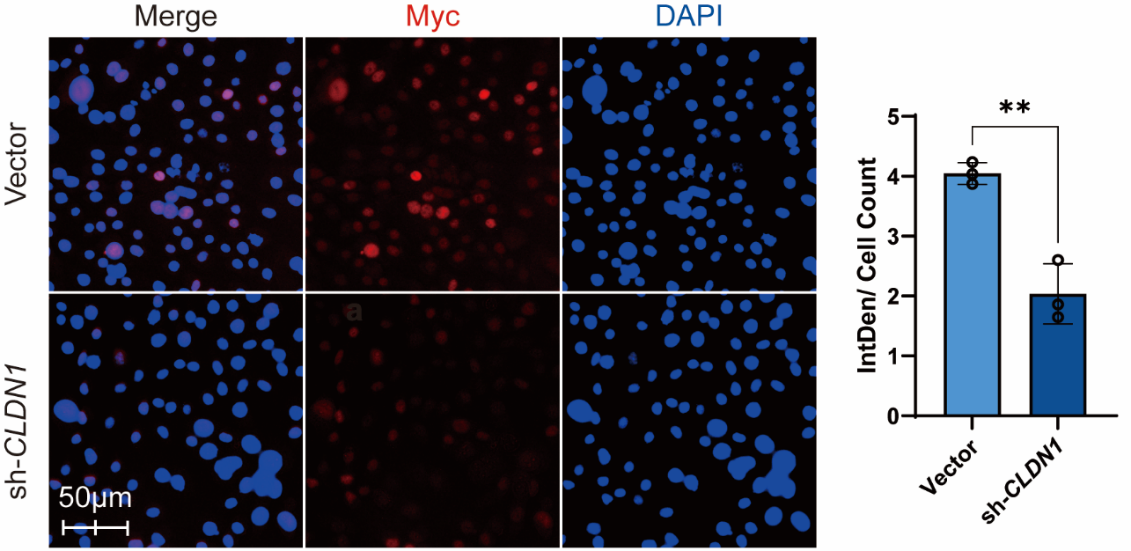


**Supplementary Figure 2****.** Immunocytochemistry of Myc expression in sebocytes. Immunocytochemistry assay showing Myc protein nuclear localization and expression in Vector and sh-*CLDN1*. Bar graphs are presented with mean ± SD. ***p*<0.01

## Supplementary Tables

**Table S1.** Basic information of participants providing skin samples for histological study and scRNA-seq.

| ID | Gender | Age | ^#^Location | Hurley stage | IHS4 score |
| --- | --- | --- | --- | --- | --- |
| Healthy controls (n=8) | | | | | |
| H1* | M | 38 | Axilla | NA | NA |
| H2* | M | 35 | Axilla | NA | NA |
| H3* | F | 28 | Axilla | NA | NA |
| H4 | M | 32 | Groin | NA | NA |
| H5 | M | 39 | Groin | NA | NA |
| H6 | M | 40 | Axilla | NA | NA |
| H7 | F | 36 | Axilla | NA | NA |
| H8 | F | 37 | Axilla | NA | NA |
| Patients with HS (n=12) | | | | | |
| P1* | M | 40 | Axilla | Ⅱ | 8 |
| P2* | M | 26 | Axilla | Ⅱ | 8 |
| P3* | F | 23 | Axilla | Ⅲ | 14 |
| P4 | M | 45 | Groin | Ⅲ | 16 |
| P5 | F | 47 | Groin | Ⅲ | 12 |
| P6 | F | 48 | Axilla | Ⅱ | 6 |
| P7 | M | 37 | Axilla | Ⅱ | 5 |
| P8 | M | 23 | Axilla | Ⅰ | 3 |
| P9 | M | 45 | Groin | Ⅱ | 6 |
| P10 | M | 38 | Axilla | Ⅰ | 5 |
| P11 | F | 18 | Axilla | Ⅰ | 2 |
| P12 | F | 30 | Axilla | Ⅰ | 5 |

^#^Anatomical location where the skin samples were taken; *H1-H3, P1-P3 were scRNA-seq sample donors, each providing one NLS and one LS sample in the same anatomical location. P1-P12 have provided LS samples for histological study, while P1-P6 have also provided NLS samples in same location with LS for histological study; NA, not applicable.

**Table S2.** Nucleotide sequences for primers used in qPCR.

| Gene | Forward Primer | | Reverse Primer |
| --- | --- | --- | --- |
| *CLDN1* | CCAGTCAATGCCAGGTACGA | ACAGCAAAGTAGGGCACCTC | |
| *MUC1* | TGCTTACAGCTACCACAGCC | GCTGGGCACTGAACTTCTCT | |
| *KRT7* | ACTGGTGGCAGTAGCAGTG | TGCGGTCCGGATGGAATAAG | |
| *IVL* | CCACTTATTTCGGGTCCGCT | CTGAGGTTGGGATTGGGGTC | |
| *KRT10* | TGGCAACTCAAGCCAGAGAG | AGCCTGGCATTGTCGATCTG | |
| *BLIMP1* | CAATGCACCTCCCTCCACTT | AGCGGTTGACGAGTGATTGT | |
| *KRT14* | CCAGAGATGTGACCTCCTCC | CTCAGTTCTTGGTGCGAAGG | |
| *GAPDH* | CTGCCAACGTGTCAGTGGTG | TCAGTGTAGCCCAGGATGCC | |

**Table S3.** *p*-value for paired comparison in Fig 3B

| Gene | HC-NLS | HC-LS | NLS-LS |
| --- | --- | --- | --- |
| *TJP1* | 0.3 | 0.3 | *p*< 0.001 |
| *OCLN* | 0.69 | 0.13 | 0.69 |
| *CLDN7* | 0.0016 | 0.0049 | 0.082 |
| *CLDN1* | *p*< 0.001 | *p*< 0.001 | 0.14 |

**Table S4.** *p*-value for paired comparison in Fig 5F

| Gene | HC-NLS | HC-LS | NLS-LS |
| --- | --- | --- | --- |
| *MUC1* | 0.0014 | 0.96 | 0.076 |
| *KRT7* | *p*< 0.001 | *p*< 0.001 | *p*< 0.001 |
| *KRT10* | *p*< 0.001 | *p*< 0.001 | *p*< 0.001 |
| *KRT1* | *p*< 0.001 | *p*< 0.001 | *p*< 0.001 |
